# MATN4 as a target gene of HIF-1α promotes the proliferation and metastasis of osteosarcoma

**DOI:** 10.18632/aging.205941

**Published:** 2024-06-17

**Authors:** Lu Zhang, Yujie Pan, Feng Pan, Songsong Huang, Fengyan Wang, Zhirui Zeng, Houping Chen, Xiaobin Tian

**Affiliations:** 1 School of Clinical Medicine, Guizhou Medical University, Guiyang 550000, Guizhou, China; 2 Department of Orthopedics, The Affiliated Hospital of Guizhou Medical University, Guiyang 550000, Guizhou, China; 3 Department of Traumatic Orthopedics, The Affiliated Hospital of Guizhou Medical University, Guiyang 550000, Guizhou, China; 4 Department of Bone and Joint Surgery, Beijing Jishuitan Hospital Guizhou Hospital, Guiyang 550000, Guizhou, China; 5 Department of Pathology, The Afflicted Hospital of Guizhou Medical University, Guiyang 550000, Guizhou, China; 6 Transformation Engineering Research Center of Chronic Disease Diagnosis and Treatment, Guizhou Medical University, Guiyang 550000, China; 7 Department of Orthopedics, Guiyang Maternal and Child Health-Care Hospital, Guiyang 550000, China

**Keywords:** HIF-1α, MATN4, metastasis, osteosarcoma

## Abstract

Background: Osteosarcoma is a highly malignant bone tumor that exhibits rapid growth and early metastasis. Hypoxia plays a pivotal role in promoting the proliferation and metastasis of osteosarcoma through a series of molecular events, which are partially mediated and regulated by HIF-1α. However, the regulatory network associated with HIF-1α in osteosarcoma remains limited. Therefore, the objective of this study was to identify critical hypoxia-associated genes and investigate their effects and molecular mechanisms in osteosarcoma cells.

Methods: Through bioinformatics analysis, matrilin-4 (MATN4) was identified as a crucial gene associated with hypoxia. The expression of MATN4 and HIF-1α was assessed using immunohistochemistry, RT-qPCR, and western blotting. The proliferative capacity of osteosarcoma cells was assessed through the utilization of CCK-8, EDU staining, and colony formation assays. The effects of MATN4 on the mobility of OS cells were evaluated using wound-healing assays and transwell assays. The interaction between MATN4 and HIF-1α was detected through chromatin immunoprecipitation.

Results: MATN4 is overexpressed in osteosarcoma tissue and cells, particularly in osteosarcoma cells with high metastatic potential. Knockdown of MATN4 inhibits the proliferation, migration, and invasion abilities of osteosarcoma cells and reverses the promoting effects of hypoxia on these functions. Additionally, HIF-1α binds to MATN4 and upregulates its expression. Interestingly, knockdown of HIF-1α reduces the stimulatory effects of MATN4 overexpression on the proliferation, migration, and invasion of osteosarcoma cells under hypoxic conditions.

Conclusions: Taken together, our results suggest that MATN4 is regulated by HIF-1α and confers a more aggressive phenotype on OS cells. This evidence suggests that MATN4 may act as a potential target for OS diagnosis and treatment.

## INTRODUCTION

Osteosarcoma (OS), the most common primary malignant bone tumor, represents 3–6% of childhood cancers and less than 1% of adult cancers [1, 2]. It is also the third most common type of tumor affecting children and adolescents after lymphomas and brain tumors, accounting for 10% of solid tumors developing in 15–19-year-old individuals [3]. The peak incidence of OS has a typical bimodal age distribution, during young growing adolescents and then between the sixth and seventh decade of life [4]. The 5-year survival rate has been improved dramatically by introduction of multiagent chemotherapy several decades ago, but is only 20% among patients presenting with metastases or recurrent disease. Metastasis to the lungs is observed in around 15% of patients with newly diagnosed osteosarcoma [5]. Furthermore, it is estimated that 60% of patients without obvious metastasis at the time of initial examination present micro-metastasis. Whereas, the prognosis of osteosarcoma patients is determined by metastasis, especially status of metastasis to the lungs [6].

Hypoxia is an inherent factor in the microenvironment of most solid tumors, including OS and is responsible for copious amounts of tumor adaptive responses. Hypoxia-related molecular events are mediated primarily by hypoxia-inducible factor 1 (HIF-1), a heterodimeric transcription factor composed of a constitutive β-subunit (HIF-1β) and an oxygen-dependent α-subunit (HIF-1α) [7, 8]. The protein level of HIF-1α is regulated by oxygen concentrations. Under normoxia, HIF-1α is hydroxylated by oxygen-dependent proline hydroxylase, and then binds to Von Hippel Lindau (VHL) protein for ubiquitination and degradation. Hypoxia prevents HIF-1α hydroxylation and subsequent ubiquitination by inhibition of prolyl hydroxylase activity, thus stabilizing HIF-1α [9, 10]. HIF-1α, as the most typical gene transcription induced by hypoxia, is overexpressed in various types of human cancers. There is compelling evidence supporting its role in tumor progression, angiogenesis and metastasis, which are often associated with negative overall survival rates and poor prognosis [11, 12]. For example, Guan et al. showed that the hypoxia-induced HIF-1α/CXCR4 pathway plays a promoting role in the proliferation and metastasis of osteosarcoma [13]. Yang et al. have found that elevation of HIF-1α correlates significantly with metastasis [14]. Unfortunately, the specific mechanism of HIF-1α regulatory network remains completely unclear.

The matrilins are a family of extracellular matrix proteins composed of four members, each with a multi-subunit structure containing von Willebrand factor type A-like domains, EGF-like domains, and an α-helical coiled-coil domain [15]. They can interact with various extracellular matrix (ECM) components, such as proteoglycans and collagens, and participate in assembly of filamentous networks within the ECMs of various tissues [16]. Studies have shown that matrilin-3 induces the expression of the pro-inflammatory cytokines, such as TNFα, IL-1, IL-6, and nitric oxide synthase (iNOS), which contribute to this matrix-specific feed-forward mechanism of cartilage degradation in osteoarthritis [17]. Recently, there has been growing interest in the relationship between matrilins and tumors. For example, Expression of matrilin-2 was up-regulated in hepatocellular carcinoma and Sporadic pilocytic astrocytoma [18]. However, up to date, the role of MATN 4 in OS has been poorly studied.

Herein, MATN 4 was identified as a key hypoxia-associated and metastasis-associated gene using bioinformatics analysis. Further, our studies demonstrated that MATN4 is a target gene of HIF-1α and promotes the proliferation, migration and invasion of OS cells under normoxia and hypoxia. These findings reveal a previously unknown link between MATN4 and HIF-1α in OS, and suggest that the MATN4 protein could serve as a novel biomarker and therapeutic target for OS.

## RESULTS

### MATN4 expression is associated with metastasis in OS

**Figure 1:**
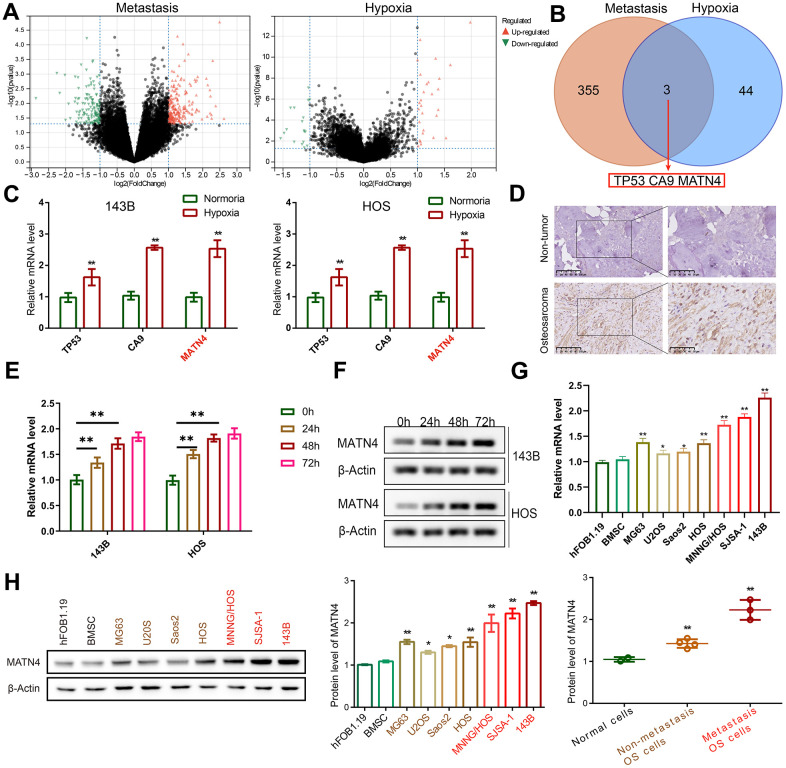
**MATN4 was involved in OS cell metastasis.** (**A**) Volcano plots were used to exhibit the differentially expressed genes (DEGs) related to metastasis and hypoxia, based on gene expression profiles obtained from the Target database. (**B**) An intersection of DEGs: TP53, CA9, and MATN4. (**C**) Expression levels of TP53, CA9 and MATN4 in 143B and HOS cells were measured by RT-qPCR under normoxia or hypoxia. (**D**) IHC was used to exhibit protein expression level of MATN4 in OS tissue and non-cancerous bone tissue. (**E**, **F**) Western blotting and RT-qPCR to measure MATN4 mRNA and protein expression under varying hypoxic conditions. (**G**, **H**) Western blotting and RT-qPCR to measure the MATN4 protein levels in normal cells (hFOB1.19 and BMSC), in OS cells with low-metastatic ability (MG63, U2OS, Saos2, HOS), and OS cells with strong metastatic ability (MNNG/HOS, SJSA-1, 143B). *, p <.05; **, p <.01.

DEGs related to hypoxia and metastasis were identified using gene expression profiles from Target database. We found 358 DEGs related to metastasis and 47 DEGs related to hypoxia in OS samples (Figure 1A), with an intersection of 3 DEGs—TP53, CA9, and MATN4 (Figure 1B). Subsequently, we validated our results by detecting the mRNA expression of TP53, CA9 and MATN4 using RT-qPCR. Our findings showed that the mRNA expression of TP53, CA9 and MATN4 was significantly up-regulated under hypoxic conditions in both 143B and HOS cells (Figure 1C). Previous research has extensively examined the involvement of TP53 and CA9 in OA. Consequently, the present study aims to investigate the role of MATN4 [19–21]. We then performed IHC to detect the protein expression of MATN4 in OS tissue and adjacent non-cancerous bone tissue. Results showed that the protein expression of MATN4 was significantly upregulated in OS tissue compared to non-cancerous bone tissue (Figure 1D). In order to assess the potential correlation between hypoxic conditions and the expression of MATN4 mRNA and protein, we conducted an investigation into the impact of varying durations of hypoxia on MATN4 expression in 143b and HOS osteosarcoma cell lines. Our findings indicate a progressive elevation in both mRNA and protein levels of MATN4 in response to prolonged exposure to hypoxia, with a more pronounced increase observed at approximately 24 and 48 hours of hypoxic conditions (Figure 1E, 1F). In addition, it was observed that the protein and mRNA level of MATN4 exhibited a gradual increase in both low-metastatic OS cells (MG63, U2OS, Saos2, HOS) and high-metastatic OS cells (MNNG/HOS, SJSA-1, 143B), when compared to normal cells (hFOB1.19 and BMSC), as illustrated in Figure 1G, 1H. The findings indicate a correlation between the expression of MATN4 and the metastatic behavior of OS cells, potentially mediated through a mechanism involving hypoxia.

### Knockdown of MATN4 inhibits OS cells proliferation, migration and invasion abilities under normoxia

**Figure 2:**
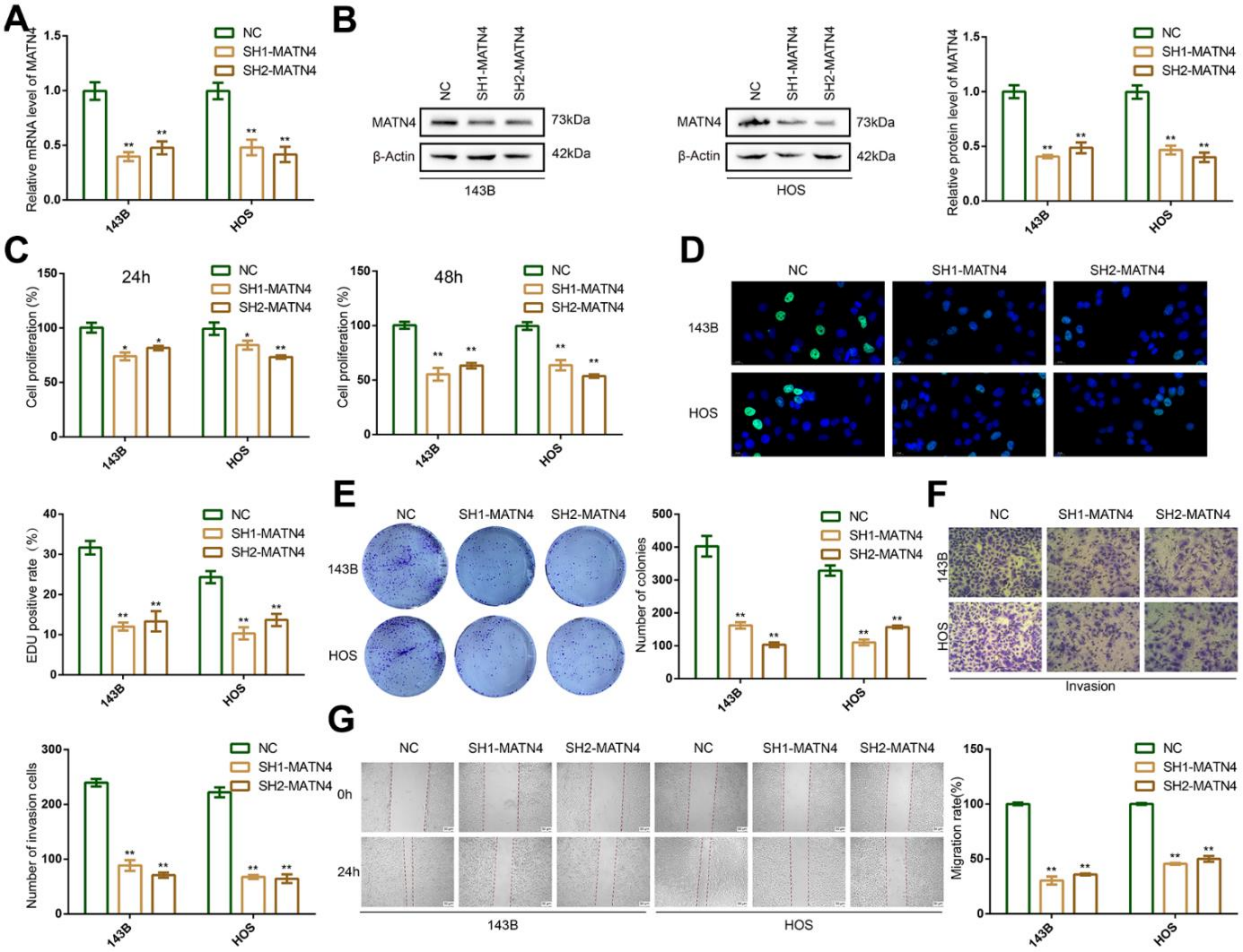
**MATN4 knockdown significantly inhibited proliferation, migration and invasion abilities of OS cells under normoxia.** (**A**, **B**) Confirmation of MATN4 knockdown by RT-qPCR and western blotting in 143B and HOS cells. (**C**, **D**) CCK-8 and EDU staining assays were performed to detect the proliferation ability of sh-scramble and SH-MATN4 OS cells at 24h and 48h. (**E**) Colony formation assays were performed in sh-scramble and SH-MATN4 OS cells. (**F**) Transwell assays were performed to detect invasion ability of OS cells after MATN4 knockdown. (**G**) Wound healing assays were used to detect migration ability of OS cells after MATN4 knockdown. *, p <.05; **, p <.01.

To investigate the relationship between expression of MATN4 and proliferation and migration of OS cells, we used two targeted shRNAs to suppress the expression of MATN4 in 143B and HOS cells. The efficiency of knockdown of MATN4 was confirmed by RT-qPCR and western blotting, both of which showed obvious inhibition of the mRNA and protein levels of MATN4 under normoxia (Figure 2A, 2B). The CCK-8 assay results demonstrated that the suppression of MATN4 expression significantly impeded the proliferation of 143B and HOS cells after 24 and 48 hours, respectively, in a normoxic environment (Figure 2C). Furthermore, EDU assay showed knockdown of MATN4 had a lower EDU positive rate, indicating lower proliferation compared to normal cells under normoxia (Figure 2D). The reduction of MATN4 expression resulted in a notable decline in the formation of colonies in both 143B and HOS cells, as depicted in Figure 2E. These findings imply that the suppression of MATN4 could potentially impede the proliferation of osteosarcoma cells. Following this, we conducted transwell and wound healing assays, which revealed a notable decrease in the invasion and migration capacity of OS cells under normoxia upon knockdown of MATN4 (Figure 2F, 2G). These results indicate that the suppression of MATN4 leads to a reduction in the proliferation, migration, and invasion capabilities of OS cells under normoxic conditions.

### Inhibition of MATN4 significantly reverses the promoting effects of hypoxia on the proliferation, migration and invasion of osteosarcoma cells

**Figure 3:**
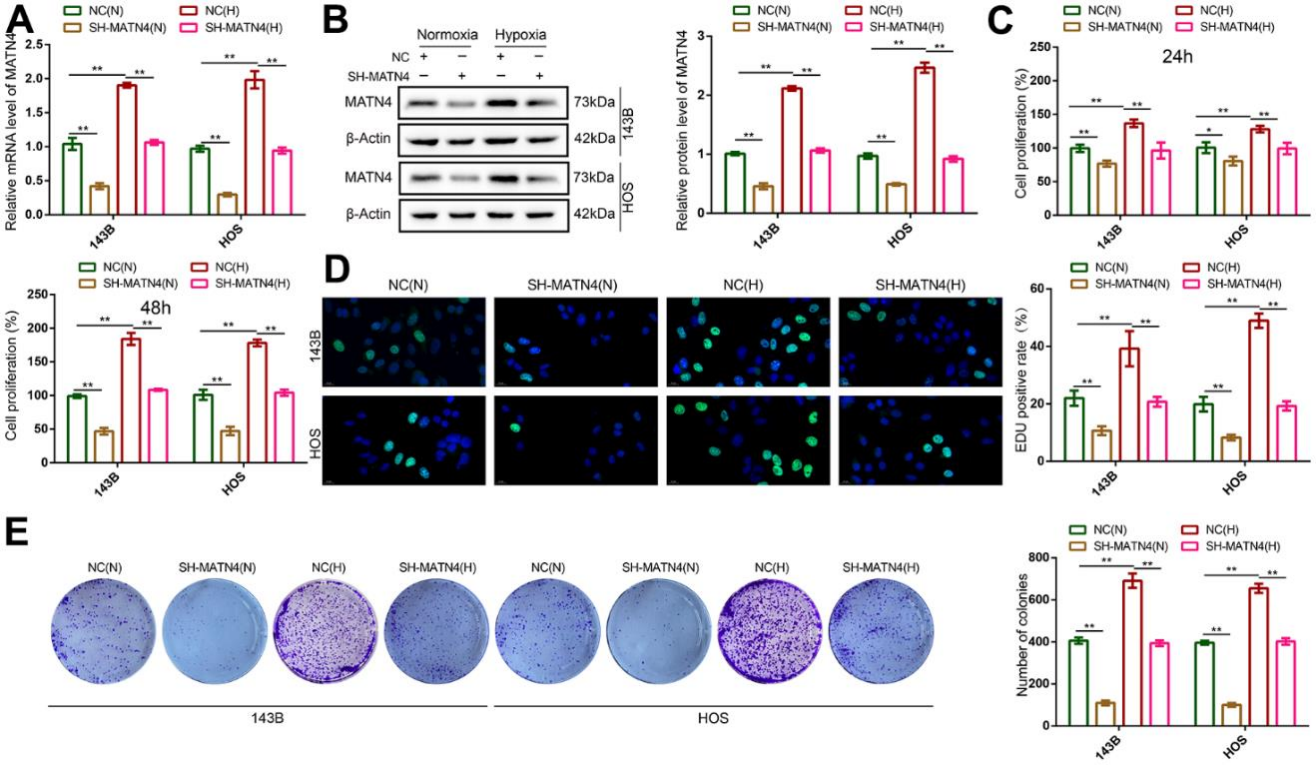
**Inhibition of MATN4 significantly reversed the promoting effects of hypoxia on the proliferation ability of OS cells.** (**A**, **B**) RT-qPCR and western blotting were used to detect mRNA and protein expression of MATN4 in OS cells transfected with sh-scramble or SH-MATN4 under normoxia and hypoxia. (**C**, **D**) CCK-8 and EDU assays were used to detect the proliferation ability of OS cells transfected with sh-scramble or SH-MATN4 at 24h and 48h under normoxia and hypoxia. (**E**) Colony formation assays were used to detect the colony formation ability of OS cells transfected with sh-scramble or SH-MATN4 at 24h and 48h under normoxia and hypoxia. *, p <.05; **, p <.01.

To elucidate the role of MATN4 inhibition in the impact of hypoxia on proliferation and migration, we conducted transfection of 143B and HOS OS cells with sh-scramble and SH-MATN4, followed by culturing under both normoxic and hypoxic conditions. We found that MATN4 mRNA and protein expression was up-regulated under hypoxia, and SH-MATN4 significantly inhibited hypoxia-induced MATN4 expression (Figure 3A, 3B).

**Figure 4:**
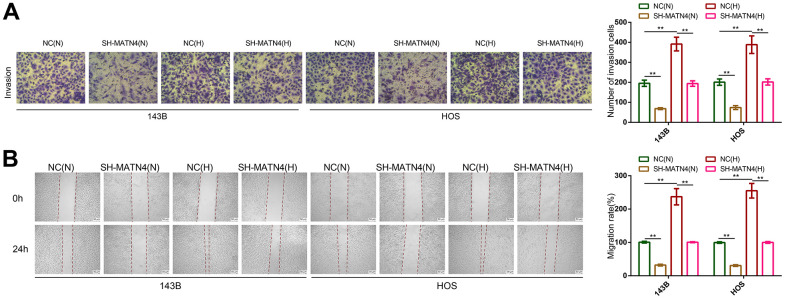
**Inhibition of MATN4 significantly reversed the promoting effects of hypoxia on the migration and invasion abilities of OS cells.** (**A**) Transwell assays were used to detect the invasion ability of OS cells transfected with sh-scramble or SH-MATN4 under normoxia and hypoxia. (**B**) Wound healing assays were used to detect the invasion ability of OS cells transfected with sh-scramble or SH-MATN4 under normoxia and hypoxia. *, p <.05; **, p <.01.

CCK-8 and EDU assays showed that hypoxia promoted the proliferation of 143B and HOS cells compared with normoxia, while MATN4 inhibition reversed the promoting effects of hypoxia (Figure 3C, 3D). Similarly, inhibition of MATN4 decreased the stimulatory effects of hypoxia on colony formation (Figure 3E). Additionally, we performed transwell and wound healing assays which suggested that hypoxia enhanced the ability of invasion and migration of OS cells, and knockdown MATN4 reversed the promoting effects of hypoxia on mobility (Figure 4A, 4B). These findings indicate that the inhibition of MATN4 effectively counteracts the stimulatory impact of hypoxia on the proliferation, migration, and invasion of osteosarcoma cells.

### MATN4 is co-expressed with HIF-1α and regulated as a target gene of HIF-1α

**Figure 5:**
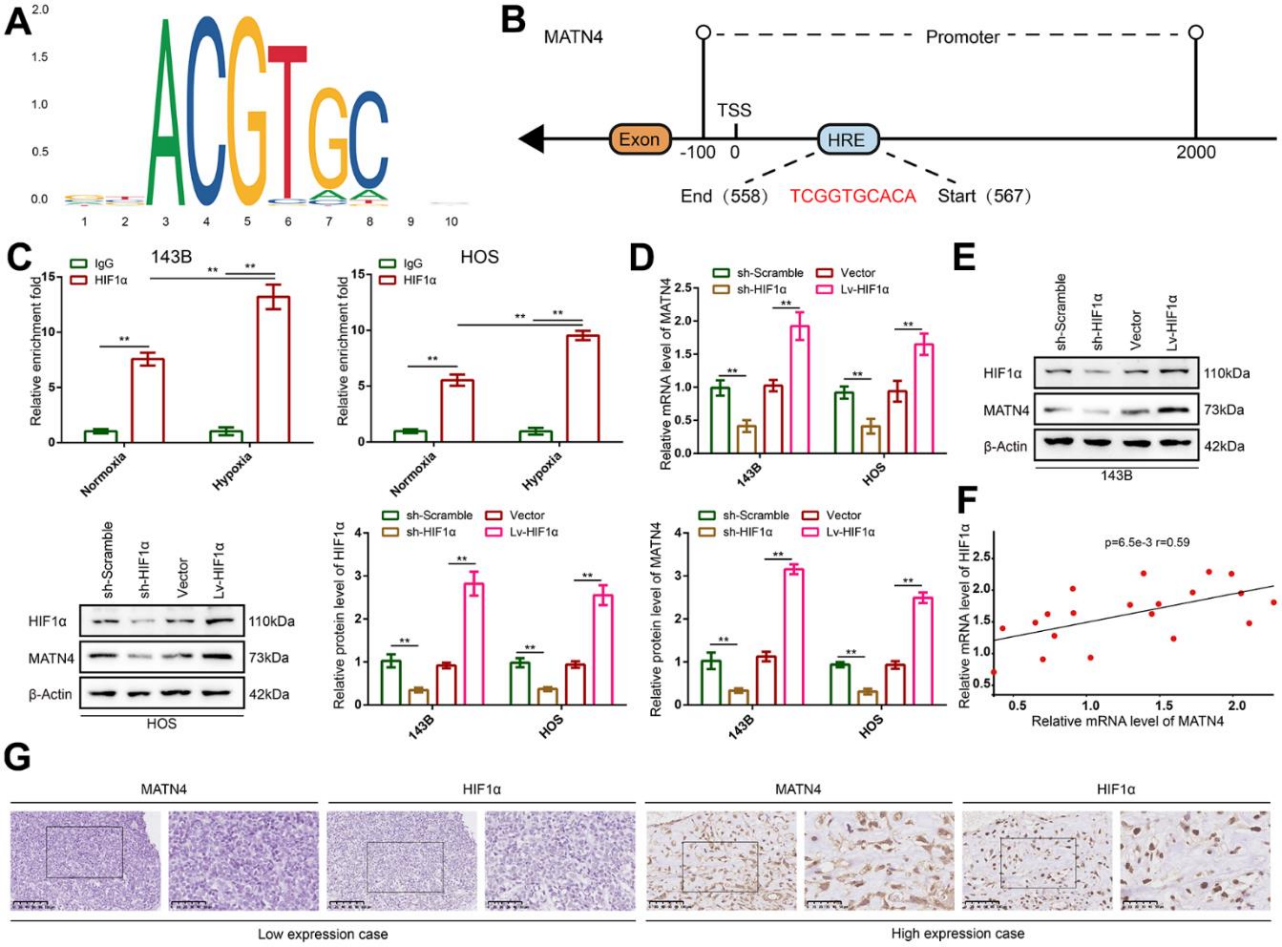
**MATN4 was co-expressed with HIF-1α and regulated as a target gene of HIF-1α in OS cells and tissue.** (**A**) Motif of HIF-1α. (**B**) Potential binding site of HIF-1α in the MATN4 promoter area. (**C**) CHIP and RT-qPCR assay were performed to verify that HIF-1α bound and up-regulated expression of MATN4 under hypoxia *in vitro*. (**D**, **E**) RT-qPCR and western blotting were performed to detect mRNA and protein expression of MATN4 in 143B and HOS cells transfected with sh-HIF1α or Lv-HIF1α under hypoxia. (**F**) Pearson correlation analysis was performed to demonstrate co-expression of HIF-1α and MATN4. (**G**) IHC assays were performed to show co-expression HIF-1α and MATN4 *in vivo*. *, p <.05; **, p <.01.

HIF-1α is a key gene that is often induced by hypoxia and plays a central role in regulating complex gene network, so we speculated that MATN4 was directly regulated by HIF-1α. HIF-1α motif (Figure 5A) and the sequence of hypoxia-responsive element (HRE) in the promoter of MATN4 were obtained from JASPAR. By comparing the HIF-1α motif to sequence of HRE, a potential binding site (558 to 567) in the MATN4 promoter area was predicted to be bound by HIF-1α (Figure 5B). In order to verify this, we used a pair of specific primers for RT-qPCR to amplify this sequence from the ChIP products using anti-IgG and anti-HIF1α. The results showed that the HIF-1α binding sequence in the MATN4 promoter area was significantly amplified in the ChIP products obtained with anti-HIF1α. In contrast, no amplification was observed in the ChIP products obtained with anti-IgG. This phenomenon was more pronounced under hypoxic conditions (Figure 5C). Additionally, we conducted chromatin immunoprecipitation (CHIP) experiments on HEK293 cells subjected to hypoxic conditions. The obtained experimental findings indicated that the regulatory association between MATN4 and HIF-1a was not limited to a specific cell type (Supplementary Figure 1A). Next, we knocked down and overexpressed HIF-1α with sh-HIF1α and Lv-HIF1α respectively. The results showed that HIF-1α knockdown significantly decreased the expression of MATN4 mRNA and protein in 143B and HOS cells under hypoxia, whereas overexpression of HIF-1α increased the expression of MATN4 mRNA and protein (Figure 5D, 5E). Then, Pearson relation analysis was used to demonstrate co-expression of HIF-1α and MATN4 in 20 OS samples and the coefficient of correlation (r-value) was 0.59 (Figure 5F). Furthermore, IHC assays were conducted to show similar results that MATN4 was co-expressed with HIF-1α in OS tissue samples (Figure 5G). Collectively, these findings suggest that MATN4 is a target gene of HIF-1α.

### Knockdown of HIF-1α reduces the stimulatory effects of MATN4 overexpression on the proliferation, migration and invasion of osteosarcoma cells under hypoxia

**Figure 6:**
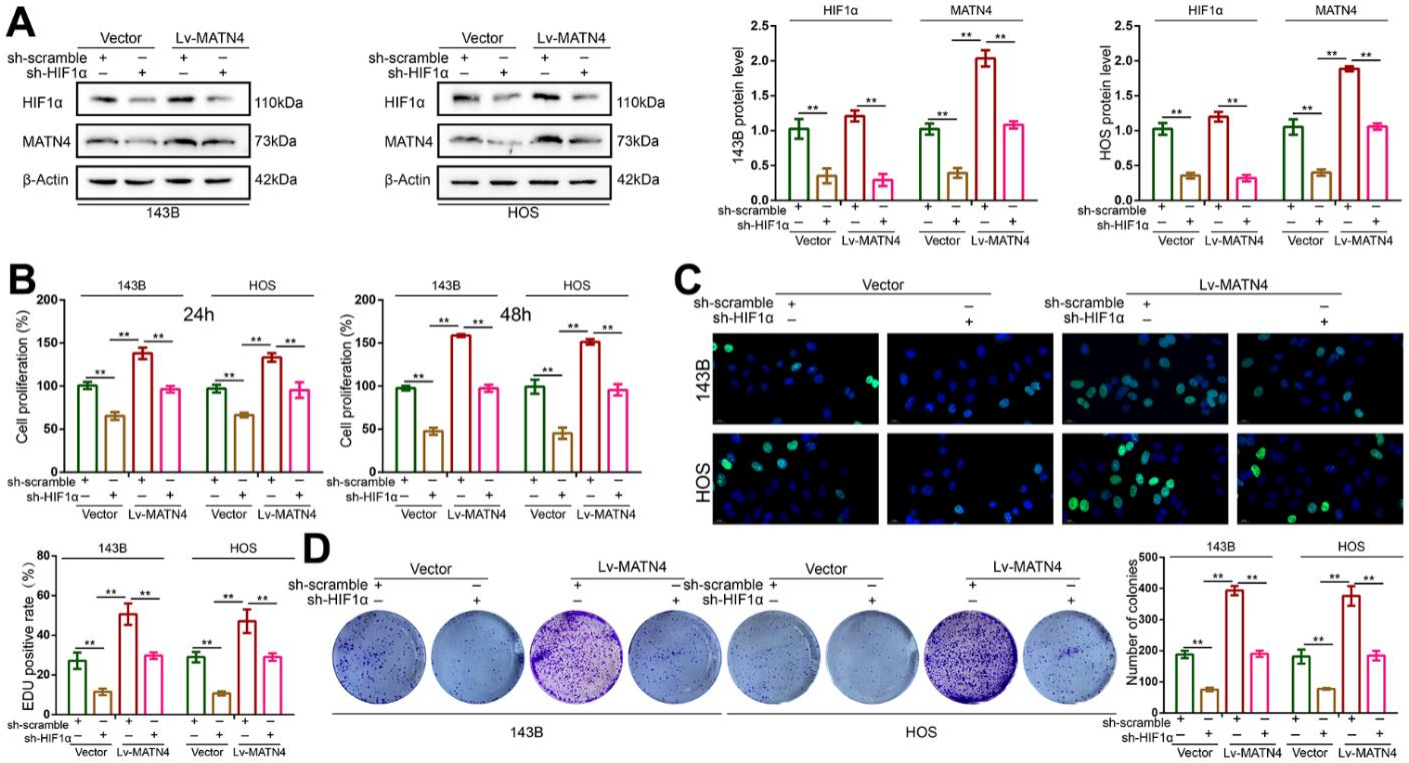
**Knockdown of HIF-1α reduced the promoting effects on proliferation of OS cell induced by MATN4 overexpression.** (**A**) Protein expression of HIF-1α and MATN4 were detected by western blotting in each group cells under hypoxia. (**B**, **C**) CCK-8 and EDU assays were used to detect the proliferation ability in each group cells under hypoxia. (**D**) Colony formation assays were used to detect the colony formation ability in each group cells under hypoxia. *, p <.05; **, p <.01.

**Figure 7:**
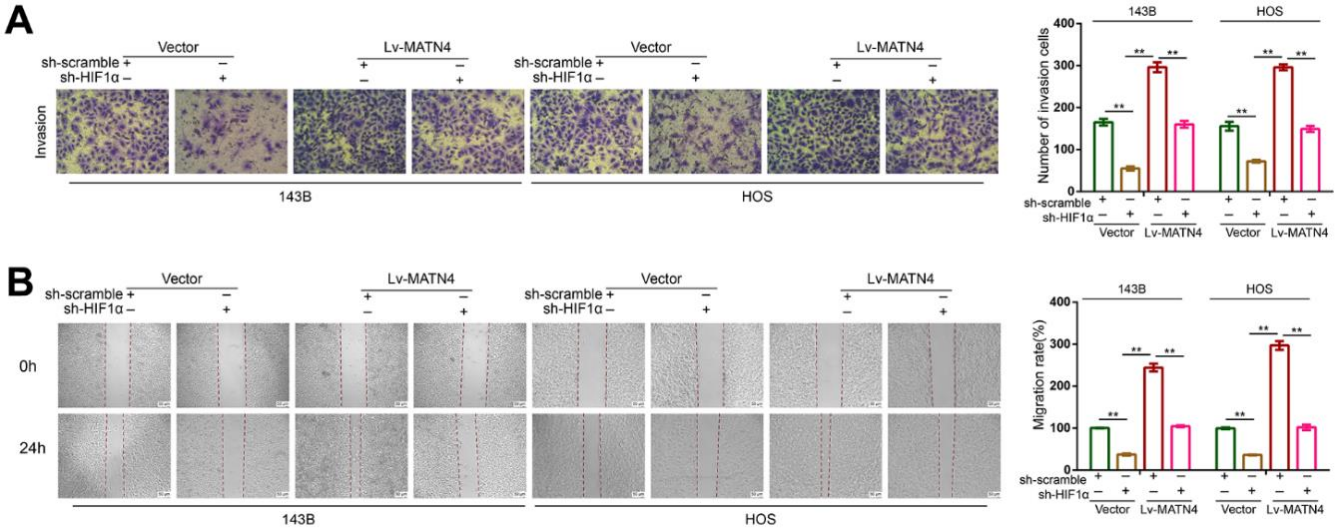
**Knockdown of HIF-1α reduced the promoting effects on migration and invasion of OS cell induced by MATN4 overexpression.** (**A**) Transwell assays were used to detect the invasion ability in each group cells under hypoxia. (**B**) Wound healing assays were used to detect the invasion ability in each group cells under hypoxia. *, p <.05; **, p <.01.

To determine whether HIF-1α participates in the MATN4 regulatory network, we transfected 143B and HOS cells with both sh-HIF 1α or sh-scramble and Lv-MATN4 or Vector. Subsequently, the protein expression of MATN4 and HIF-1α was measured by western blotting, as the results showed that knockdown of HIF-1α reduced the expression of MATN4-overexpressing OS cells under hypoxia and that overexpression of MATN4 had little effect on HIF-1α expression (Figure 6A). The results of CCK-8 and EDU assays showed that overexpression of MATN4 had a significantly promoting effect on proliferation of OS cells under hypoxia, and this effect was reduced by HIF-1α knockdown (Figure 6B, 6C). Similarly, knockdown of HIF-1α reduced stimulatory effects of MATN4 overexpression on colony formation (Figure 6D). The wound-healing and transwell assays demonstrated that knockdown of HIF-1α alleviated the promoting effects on migration and invasion of OS cells induced by MATN4 overexpression under hypoxia (Figure 7A, 7B). This evidence indicates that knockdown of HIF-1α reduces the promoting effects induced by MATN4 overexpression on the proliferation, migration and invasion of osteosarcoma cells under hypoxia.

## DISCUSSION

Osteosarcoma (OS) is the most common malignant bone-related cancer in adolescents and has a complex heterogeneity [22]. The 5-year overall survival rate for patients with metastases remains very low, below 30%, which suggests that metastasis is a strong predictor of the survival of patients with osteosarcoma [23]. Despite the diligent endeavors of researchers, the implementation of chemotherapy yielded a notable enhancement in the 5-year survival rate, yet it failed to elicit any substantial alteration in overall survival (OS) subsequently [24]. This suggests that we require a deeper understanding of the mechanisms underlying the metastasis of OS. The previous studies have shown that hypoxia confers a more aggressive phenotype on OS cells, including higher abilities of proliferation and metastasis, by activating a cascade of molecular events partly mediated and regulated by HIF-1α [25, 26]. Although the HIF-1α related regulatory network and its effects under hypoxia have been partially revealed, the network in OS is still limited. Herein, we enriched the downstream of regulatory network in HIF-1α induced by hypoxia.

Matrilins, as a member of non-collagenous extracellular matrix proteins, are believed to act as adaptors in the ECM assembly, and the interactions between matrilins and ECM have been extensively investigated. Previous research has shown that matrilin-1 and matrilin-3 are mainly expressed in skeletal tissue, whereas matrilin-2 and matrilin-4 have a more widespread expression pattern [15]. These findings imply the biological functions of matrilin-family has obvious tissue specificity. For example, Malin et al. observed wild type mice up-regulated the expression of matrilin-2 after peripheral nerve injury and matrilin-2-deficient mice had an inferior recovery after injury, suggesting a role for matrilin-2 in axonal growth [27]. Uckelmann et al. demonstrated that matrilin-4 regulates HSC proliferation and expansion in response to inflammatory stress, chemotherapy, and transplantation via CXCR4 signaling [28]. In recent years, there has been a growing body of research focused on elucidating the role of matrilins in tumorigenesis. According to Wang and Zhang et al.’s bioinformatics analysis, the expression of matrilin-3 was found to be markedly elevated in Gastric cancer which suggested that matrilin-3 was related to cancer development and inferior prognostic results [29, 30]. Vincourt et al. found that matrilin-3 is dramatically increased and improperly localized in cartilaginous tumors, which downregulates transcription factors SOX9 through epidermal growth factor domain 1-dependent signaling [31]. However, the effect of MATN4 on tumors, especially osteosarcoma, has rarely been studied.

In our study, we found that MATN4 was related to both metastasis and hypoxia through bioinformatics analysis. Subsequently, we proceeded to authenticate its expression in both osteosarcoma cells and tissues, thereby implying a potential correlation between the expression magnitude of MATN4 and the metastatic potential of osteosarcoma. Notably, MATN4 exhibited a substantial increase in osteosarcoma cells with a pronounced ability to metastasize, indicating its potential contribution to the proliferation and metastasis of osteosarcoma. Consequently, the downregulation of MATN4 resulted in a significant inhibition of proliferation, migration, and invasion in OS cells under normoxic conditions. Then we investigated the association between MATN4 and hypoxia by comparing the alterations in MATN4 expression, proliferation, and mobility between MATN4 knockdown OS cells and normal OS cells under both normoxic and hypoxic conditions. The findings from this analysis demonstrated that hypoxia upregulated the expression of MATN4 and induced a more aggressive phenotype in OS cells, which was effectively counteracted by the knockdown of MATN4. By comparing HIF-1α motif to the sequence of HRE in the promoter of MATN4, we speculated that HIF-1α might mediate hypoxia-induced expression of MATN4. Indeed, we found HIF-1α bound to promoter area and regulated the expression of MATN4 in OS cells via RT-qPCR, western blotting and CHIP assays. Similarly, IHC assays showed that MATN4 was co-expressed with HIF-1α in OS tissue. Furthermore, to determine whether MATN4 is involved in the biological function of OS cells induced by HIF-1α under hypoxia, we performed knockdown of HIF-1α and overexpression of MATN4 using different combinations. These results indicated that knockdown of HIF-1α reduces the stimulatory effects of MATN4 overexpression on the proliferation, migration and invasion of osteosarcoma cells under hypoxia.

Previous studies have provided limited understanding of the signaling pathways involving matrilins, which does not support further investigation into the molecular mechanism of MATN4 on OS cells. There are only a few relevant research studies as follows: Fullár et al. indicated that lack of matrilin-2 contributes to development of hepatocellular carcinoma via Erk1/2 and GSK-3β pathways *in vivo* [32]. Mann et al. showed that matrilins, with the exception of MATN4, bind to integrins. The interaction between matrilins and integrins is relatively weak, and it is unclear whether this interaction can activate signal transduction and induce gene expression [33]. Klatt et al. proposed that matrilin-3, like collagen II, may activate the mitogen activated protein kinase (MAPK) cascade and NF-κB signaling via the discoidin domain receptor 2 [34]. Therefore, more subsequent studies should be performed to reveal the regulatory network associated with MATN4.

In summary, we have uncovered a novel link between MATN4 and HIF-1α whereby MATN4 is regulated by HIF-1α and confers a more aggressive phenotype on OS cells. Thus, MATN4 may contribute to be a potential target for OS diagnosis and treatment.

## MATERIALS AND METHODS

### Cell lines and cell culture

Human normal osteoblasts (hFOB1.19), human bone marrow-derived mesenchymal stem cells (BMSC), human osteosarcoma cell lines MG63, U2OS, Saos2, HOS, MNNG/HOS, SJSA-1, 143B were obtained from American Type Culture Collection (Manassas, VA., USA). All cells were cultured in Dulbecco’s Modified Eagle’s Medium (DMEM; Gibco, NY, USA), supplemented with 10% fetal bovine serum (FBS; Gibco, NY, USA) and incubated in a humidified atmosphere at 37° C, under 5% CO_2_. The normoxic condition was set at 21% oxygen, while the hypoxic condition was set at 1% oxygen [35, 36].

### Clinical sample collection and tissue ethics

All patients had not received chemotherapy and radiotherapy before tissue collection. Out of 20 OS patients, 10 patients had been diagnosed with stage I–II (Enneking staging system) and the remaining 10 were had been diagnosed with stage II–IV (Enneking staging system). All samples were stored at -80° C until used for experiments.

### Bioinformatics analysis

Gene expression profiles, including 88 OS samples, were obtained from the TARGET database. Differentially expressed genes (DEGs) related to hypoxia and metastasis were identified via the “limma” package of R software [37, 38]. |logFC| ≥ 1 and adjusted P-value < 0.05 were considered significant. DEGs were visualized using a volcano plot, and the overlapping DEGs between the two sets were compared for further analysis. Moreover, the HIF-1α motif was obtained from JASPAR (http://jaspar.genereg.net/) and statistical significance was set at P-value < 0.05.

### Quantitative real-time PCR (RT-qPCR)

Total RNA was extracted from cultured cells using TRIzol reagent (Yeasen, Shanghai, China), and the concentration of total mRNA was determined by a nanodrop spectrophotometer. First-strand cDNA was synthesized with the PrimeScript™ RT Reagent (Thermo Fisher Scientific, Waltham, MA, USA) according to the manufacturer’s protocol. Quantitative RT-PCR amplification of target genes was performed using SYBR Green Abstract One Step RT-PCR Mix (Sangon Biotech, Wuhan, China) and using the StepOnePlus Real-Time PCR system (Applied Biosystems, Waltham, MA, USA) according to the manufacturer’s protocol, with β-actin as the reference gene. The primers used for RT-qPCR were as follows:

CA9 forward: 5ʹ-CTCCTGGGCTAGAGATGGCT-3ʹ;

CA9 reverse: 5ʹ-CCAAGGCCTCGTCAACTCTG-3ʹ;

Tp53 forward: 5ʹ-TCATACTGCTGAGCACTCCAA-3ʹ;

Tp53 reverse: 5ʹ-TCAGAACCGAGTGCATGTGAA-3ʹ;

MATN4 forward: 5ʹ-GTCCCCCAAATCAGTGGAGG-3ʹ;

MATN4 reverse: 5ʹ-GGTGTGGGGTAAGTGACTCG-3ʹ;

HIF1α forward: 5ʹ-GAACGTCGAAAAGAAAAGTCTCG-3ʹ;

HIF1α reverse: 5ʹ-CCTTATCAAGATGCGAACTCACA-3ʹ;

β-actin forward: 5ʹ-CATGTACGTTGCTATCCAGGC-3ʹ;

β-actin reverse: 5ʹ-CATGTACGTTGCTATCCAGGC-3ʹ.

### Immunohistochemistry (IHC)

The expression of MATN4 and HIF-1α was detected by immunohistochemistry in OS tissue. Briefly, OS tissue slices that had been fixed with formaldehyde polymer and embedded in paraffin were successively deparaffinized in xylene and rehydrated in a gradient of ethanol. Antigen retrieval was performed by citrate salts (pH 6.0). Slices were incubated with 3% H_2_O_2_ in order to inactivate endogenous peroxidase activity and then blocked by 5% bovine serum albumin. Primary antibodies to HIF-1α (1:100; Cat No: 20960-1-AP, Proteintech, China) and MATN 4 (1:50; Cat No: BS71761, Bioworld, China) were incubated with sections at 4° C overnight. After washed by phosphate-buffered saline (PBS), the sections were incubated with corresponding secondary antibodies for 1 hour, and then stained with DAB.

### Western blotting

The cell lysates were prepared using RIPA lysis buffer (Nanjing Jiancheng Bioengineering Institute, Nanjing, China) supplemented with 1/100 phenylmethanesulfonyl fluoride (Nanjing Jiancheng Bioengineering Institute) as protease inhibitors. Protein concentration was then quantified using a BCA Protein Assay Kit (Thermo Fisher Scientific, Waltham, MA, USA). Protein samples were separated by sodium dodecyl sulfate-polyacrylamide gel electrophoresis (SDS-PAGE) using a 5% concentrated gel and a 12% separation gel, and then transferred onto a polyvinylidene fluoride membrane (Millipore, St Louis, MO, USA). Membranes were blocked in a TBST solution containing 5% skim milk at room temperature for 2 hours, and subsequently incubated overnight at 4° C with the appropriate primary antibodies: anti-MATN4 antibody (1:2000; Cat No: BS7176, Bioworld), anti-HIF-1α antibody (1:1000; Cat No: 20960-1-AP, Proteintech) and β-actin (1:10,000; Cat No: AC026, ABclonal, Wuhan, China). After washing with TBST for three times, the membranes were incubated with horseradish peroxidase-conjugated secondary antibodies at room temperature for 2 hours. Finally, protein bands were detected using chemiluminescence and analyzed with ImageJ. The detection of β-actin on the same membrane was used as a loading control.

### Cell transfection

Human MATN4-targeting short hairpin RNA oligonucleotide sequences (SH-MATN4), HIF-1α-targeting short hairpin RNA oligonucleotide sequences (sh-HIF1α), and corresponding scramble shRNA (sh-scramble) as negative control were designed and synthesized by GeneChem (Shanghai, China). The sequence of SH1-MATN4 and SH2-MATN4 were 5ʹ-GAGGAUUUAUGUUCUUCUA-3ʹ, 5ʹ-GAGCAAGACUCUAUCUAAA-3ʹ, respectively. The sequence of sh-HIF1α and the corresponding sh-scramble were 5ʹ-GGUCAGGAGUUCAAGACAA-3ʹ and 5ʹ-UUCUCCGAACGUGUCACGU-3ʹ, respectively. The MATN4-overexpression lentivirus (Lv-MATN4) and HIF1α-overexpression lentivirus (Lv-HIF1α) were obtained from GeneCopoeia (Rockville, MD, USA), and the empty vector (Vector) was used as a negative control. Polybrene (Thermo Fisher Scientific) and Lipo2000 (Thermo Fisher Scientific) were used for lentiviral transduction and shRNA transfection, respectively. Overexpression cell lines were selected for 2 weeks with 1.0 μg/ml puromycin after 72 h of transduction.

### Cell counting kit-8 assay

Cell counting kit-8 (Dojindo Laboratories, Kumamoto, Japan) was used to detect cell proliferation behavior of 143B and HOS. In brief, cells were seeded at a density of 3×10^3 cells per well in a 96-well plate and cultured at 37° C in 5% CO_2_ under normoxia or hypoxia. After 24 and 48 hours, medium was removed and 100 μl of fresh culture medium containing 10 μl of CCK8 reagent was added to each well, then incubated for 2 h at 37° C. Finally, OD value was detected at 450 nm wavelength in each well.

### 5-Ethynyl-2’-deoxyuridine (EDU) assay

The EDU assay was performed using a BeyoClick EdU-488 Proliferation Detection Kit (Beyotime, Shanghai, China). Briefly, while the OS cells were adhering to the 6-well plate, the primary medium was removed. Then an equal volume of fresh medium and 2X EDU working solution were added to each well and cells were cultured at 37° C for 2 hours. Fixation was performed at room temperature with 4% paraformaldehyde for 15 minutes, followed by incubation with PBS containing 0.3% Triton X-100 (Boster, Wuhan, China) at room temperature for 10 minutes. After adding 500 μl of Click Additive Solution to each well, the OS cells were incubated in a dark place for 30 minutes and then stained with DAPI for 10 minutes. The proportion of EDU-positive cells was subsequently determined using fluorescence microscopy.

### Colony formation assay

OS cells were seeded into six-well plates (1×103 cells/well) and cultured under normoxia or hypoxia. After two weeks, cells were fixed with 4% paraformaldehyde at room temperature for 30 minutes, and subsequently stained with 0.1% crystal violet dye. Colonies with an area >10 mm^2^ were counted.

### Transwell assay

Transwell assay was performed using transwell chambers (8 μm pores, BD Biosciences, Franklin Lakes, NJ, USA), which were pre-coated with Matrigel (BD Biosciences, San Jose, CA, USA). Cells undergoing logarithmic growth were digested with trypsin, and then resuspended in serum-free culture medium. Next, 200 μl of cell suspension containing 10,000 OS cells was placed into the upper chambers and added 700 μl DMEM medium containing 20% FBS to the lower chamber. Transwell plates were incubated at 37° C for 24 hours. Then, upper surface cells of chamber were wiped out, and fixed with 4% paraformaldehyde at room temperature for 20 minutes, subsequently stained with 0.1% crystal violet dye, and counted under a microscope. Three independent experiments were performed.

### Wound healing assay

OS cells were harvested and planted into 6-well plates until confluency reached 90%. Prior to scratching, cells were pretreated with 10 μg/ml mitomycin C for 30 min. Then, a 200 μL sterilized tip was used to create a wound. After washing the floating cells 3 times with PBS, FBS-free medium was added to each well. The scratches were photographed at 0 and 24 h. The percentage of the wound healing was measured using Image Pro Plus software [39].

### Chromatin immunoprecipitation (ChIP)

The ChIP assay was performed using the MAGnify™ Chromatin Immunoprecipitation System (Thermo Fisher Scientific) according to the manufacturer’s instructions. In brief, 143B and HOS cells were cultured under normoxia and hypoxia. The OS cells were crosslinked with formaldehyde to preserve the chromatin structure, incubated with Lysis Buffer containing protease inhibitors, and then disrupted by sonicator. Notably, samples should be kept cool on ice throughout the entire process to prevent protein degradation or reversal of crosslinks. After the samples were centrifuged, the supernatant was collected and then incubated with anti-HIF1α or anti-IgG overnight at 4° C. Next, the samples were immunoprecipitated using Dynabeads. After washing and reversing the crosslinking, the immunoprecipitated products were detected by RT-qPCR.

### Statistical analysis

Prism v 7.0 (GraphPad Inc., La Jolla, CA, USA) was utilized for statistical analysis. The statistical method of Pearson correlation was employed to determine the correlation between relative expression of MATN4 and HIF-1α, and significance was defined as r > 0.4 and p < 0.05. The difference within two groups was carried out using unpaired t-test. And the difference within multiple groups using one-way ANOVA, followed by a post-hoc Tukey’s test. p < 0.05 was considered statistically significant.

## Supplementary Material

Supplementary Figure 1
