# Supplementary material for: MATN4 as a target gene of HIF-1α promotes the proliferation and metastasis of osteosarcoma
Source: Aging (Albany NY). 2024 Jun 17;16(12):10462–76. doi: 10.18632/aging.205941 (PMC11236324; doi:10.18632/aging.205941)
Supplement: Supplementary Figure 1 [file aging-16-205941-s001.pdf]

SUPPLEMENTARY FIGURE

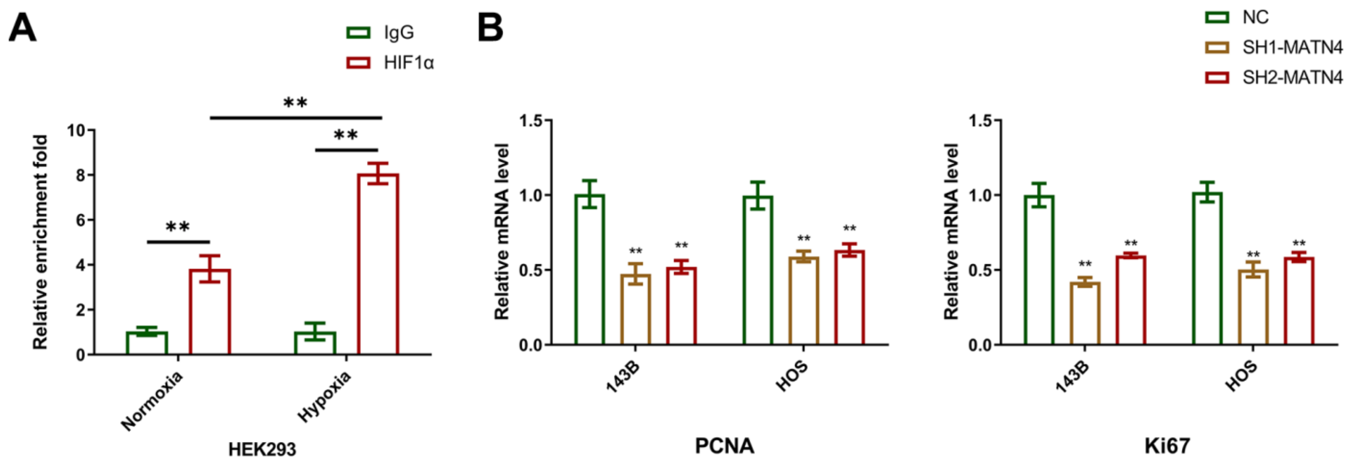

Supplementary Figure 1. (**A**) ChIP was conducted in HEK293 cells to confirm the regulatory interaction between MATN4 and HIF-1a is not exclusive to osteosarcoma cells. (**B**) Following the downregulation of MATN4, the expression levels of PCNA and Ki67 in 143B and HOS cells were quantified using RT-qPCR.
